# Intraoperative rupture of popliteal artery pseudoaneurysm secondary to distal femur osteochondroma: case report and review of the literature

**DOI:** 10.11604/pamj.2013.16.150.3510

**Published:** 2013-12-20

**Authors:** Rita Hajji, Hamid Jiber, Youssef Zrihni, Othman Zizi, Abdellatif Bouarroum

**Affiliations:** 1 Department of vascular surgery, UHC Hassan II, Fes, Maroc

**Keywords:** Exostoses, osteochondroma, popliteal artery pseudoaneurysme

## Abstract

Vascular complications from osteochondroma are rare and include essentially stenosis, occlusion, and pseudoaneurysms. The authors report an original case of intraoperative rupture of undiagnosed popliteal artery pseudoaneurysm during resection surgery for a distal femur osteochondroma.

## Introduction

Exostoses are the most frequent benign bone tumors. They account from 10 to 15% of both benign and malignant bone tumors [1, 2]. Osteochondroma may be present as a solitary lesion or in exostoses multiple hereditary form. Usually, they are asymptomatic. However, they may cause various complications such as arterial pseudo-anevrysm. This pseudo anevrysm is most often located on the popliteal artery. [3] Pseudo-anevrysm results from an arterial injury due to exostose. Its mechanism development is still unknown because it is usually asymptomatic. Our case involved a 20 year-old boy with multiple hereditary exostoses. A large pseudo-anevrysm was observed and broken during surgery. Then, He was admitted for care in our Hospital. Although arterial pseudo-anevrysms rarely develop, we must always pay attention to patients who present multiple exostoses.

## Patient and observation

**Figure 1:**
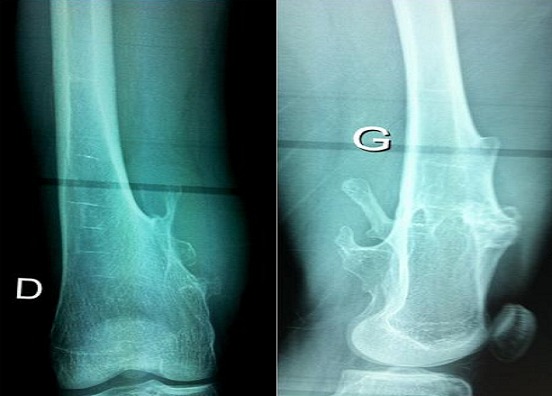
X-ray of the knee showing the bilateral exostoses of distal femur

A 22-year old man with hereditary multiple exostoses was scheduled for a surgical excision of distal right femur osteochondroma. The preoperative evaluation was unremarkable, distal pulses were presents and the leg was well perfused. Knee X-rays showed bilateral distal femoral exostosis (Figure 1)

**Figure 2:**
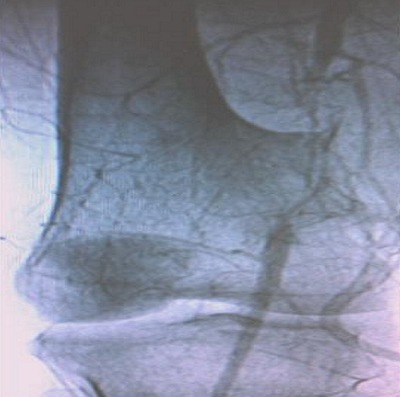
Pre-operative Digital subtraction arteriography showing the popliteal artery occlusion

**Figure 3:**
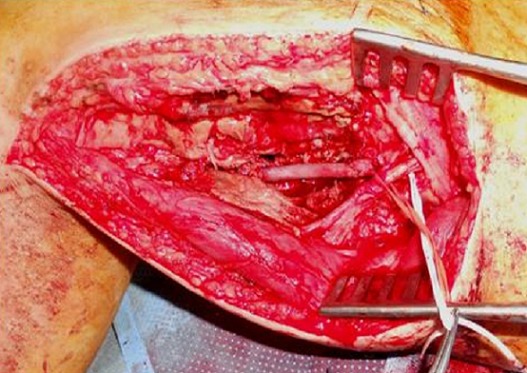
Intraoperative image: popliteal artery repaired with a reversed saphenous vein interposition graft

During intervention a massive bleeding occurred, and for hemostasis, the surgeon was forced to lie the popliteal artery. Three hours later, the patient was admitted to our emergency department with a relative ischemia of the right lower limb, with normal vital signs. On physical examination, sensation was present but reduced to the foot, the popliteal and distal pulses were absents. Laboratory tests were normal except for haemoglobin; it was at 9 g/dl. An arteriography was performed and clearly showed a popliteal artery occlusion opposite the incision (Figure 2). The patient underwent surgery. The popliteal artery was exposed through a medial supra-geniculate and infrageniculate approach. The artery was found ligated near the prior surgical site. The popliteal artery wall was repaired by veinous graft after vascular clamps were applied (Figure 3). The postoperative course was uneventful and the patient regained his normal activity. There was no sign of malignant transformation on histopathological examination of bone

## Discussion

Osteochondroma is one of the most common bone tumors. It is exceptionnaly responsible for vascular complications from which pseudo-anevrysm constitutes the most frequent entity. Hereditary multiple exostoses is a multi-phenotypic autosomal dominant disorder that affects 1 per 50000 in the general population for Caucasians [4]. It is a disorder mapped to the exostoses genes (EXT) [5]. Exostoses develop shortly after birth and continue to develop throughout childhood and puberty [6]. Malignant degeneration is the most severe complication [6].

Osteochondroma may cause various complications such as arterial pseudo-anevrysms. The vascular disease genesis is due to repeated exercices, such a s knee flexion which could chronically abrad the popliteal artery and produce an adventicial defect followed by a pseudo-anevrysm. [7]

Exostose is often asymptomatic. A chronic edema may reveal popliteal artery disease. It may also lead to many other problems: peripheral nerves compression, blood vessels, distal ischemia, phlebitis and pulsative mass [8]. In our case, the pseudo-anevrysm was revealed by the rupture which occurred during surgery: While the exostose was being removed, a false anevrysm with arterial bleeding was found. In this case report, osteochondroma was removed without arteriography.

In addition to this patient, 2 other cases of rupture of popliteal artery have been reported in the literature. The pathologic diagnosis of osteochondroma is made by radiographic examination which demonstrated an osteocartilaginous exostosis [9]. The clinical diagnosis of a false anevrysm was confirmed by a digitalized arteriography or vascular ultrasonography.

The first case is a 33 -year -old patient. Osteochondroma was located on the fibula. The rupture occurred after traumatisme.the popliteal artery was repaired by vein patch angioplasty [10]. The second case is a 20 -year -old patient; the exostose was located on the tibia. The rupture came after a sport's accident. The artery was repaired by veinous graft.

In our patient, the exostosis was removed by an orthopaedic surgeon. No vascular ultrasonography or arteriography have been realized before surgery. After removing the exostosis, bleeding occurred and a ligation of the popliteal artery was reported by the orthopeadic surgeon. A part from the surgery, there was no history of trauma that might have caused a fracture of the exostosis. Pain, edema, pulsative mass are the most frequent pseudo-anevrysm clinical signs. However, the rupture may reveal a pseudo-anevrysm not yet known. In this case, surgery is required.

Surgical repair and excision of the adjacent osteochondroma is considered as the treatment of choice. One case of successful transarterial embolization using helical microcoils in the treatment of pseudoaneurysm on osteochondroma that was located on the superficial femoral artery. The popliteal artery aren&39;t suitable to this kind of treatment. Our patient is young, the pseudoanevrysm was broken when he was admitted to our hospital and the popliteal artery was ligated by the orthopaedic surgeon. So, surgery was required to restitute a normal vascular status.

## Conclusion

Osteochondroma may be present as a solitary lesion or in the form of hereditary multiple exostoses. Vascular complications of exososes are infrequent and the association with false anevrysms is exceptional. Acute rupture is rare, In a recent review of the literature, 38 cases of popliteal pseudoaneurysm secondary to exostosis have been reported, and only two of which were ruptured. To our knowledge this is the first case of intraoperative rupture occurring during resection surgery for distal femur osteochondroma. A combined orthopaedic and vascular surgery must always be undertaken when there is exostose associated to a false anevrysme.
